# Sex-specific differences and postoperative outcomes of minimally invasive and sternotomy valve surgery

**DOI:** 10.1093/ejcts/ezab369

**Published:** 2021-08-15

**Authors:** Marco Moscarelli, Roberto Lorusso, Gianni D Angelini, Nicola Di Bari, Domenico Paparella, Khalil Fattouch, Alberto Albertini, Giuseppe Nasso, Francesca Fiorentino, Giuseppe Speziale

**Affiliations:** 1 Department of Cardiovascular Surgery, GVM Care & Research, Anthea Hospital, Bari, Italy; 2 Cardio-Thoracic Surgery Department, Heart & Vascular Centre, Maastricht University Medical Centre, Cardiovascular Research Institute Maastricht, Maastricht, Netherlands; 3 Department of Cardiovascular Surgery, Bristol Heart Institute, University of Bristol, Bristol, UK; 4 Department of Cardiovascular Surgery, GVM Care & Research, Santa Maria Hospital, Bari, Italy; 5 Department of Cardiovascular Surgery, GVM Care & Research, Maria Eleonora Hospital, Palermo, Italy; 6 Department of Cardiovascular Surgery, GVM Care & Research, Maria Cecilia Hospital, Cotignola, Ravenna, Italy; 7 Department of Surgery and Cancer and Imperial Clinical Trials Unit, Imperial College London, London, UK

**Keywords:** Minimally invasive surgery, Female sex, Postoperative mortality, Postoperative outcomes

## Abstract

**OBJECTIVES:**

Female sex is an established risk factor for postoperative complications after heart surgery, but the influence of sex on outcomes after minimally invasive cardiac surgery (MICS) for valvular replacement/repair remains controversial. We examined whether the role of sex as a risk factor varies by surgical approach [MICS vs conventional sternotomy (ST)] and further assessed outcomes among female patients including in-hospital mortality and postoperative complications by surgical approach.

**METHODS:**

We analysed data from a multicentre registry for patients who underwent isolated aortic valve and mitral surgery with MICS or ST. The primary outcome was in-hospital mortality. Propensity score matching was used to minimize between-group differences.

**RESULTS:**

Among the 15 155 patients included in the study, 7674 underwent MICS (50.6%). Female sex was equally distributed in the MICS and ST groups (47.3% vs 47.6%, respectively). Risk for surgery was higher in the ST group than in the MICS group {EuroSCORE II: 4.0 [standard deviation (SD): 6.8] vs 3.7 [SD: 6.4]; *P* = 0.005}, including among female patients only [ST vs MICS 4.6 (SD: 6.9) vs 4.2 (SD: 6.3); *P* = 0.04]. Mortality did not significantly vary by procedure among women [MICS vs ST, 2.4% vs 2.8%; hazard ratio 1.09, 95% confidence interval 0.71–1.73; *P* (surgical approach × sex) = 0.51]. The results also did not vary after adjusting for confounders.

**CONCLUSIONS:**

Female sex was associated with higher mortality in patients undergoing valve surgery, regardless of surgical approach. In female patients, MICS did not provide any benefits over ST in terms of in-hospital deaths or postoperative complications.

**Subject collection:**

117, 125.

**Figure:**
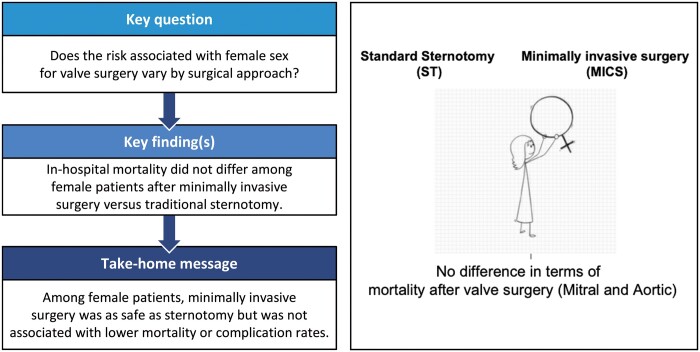

## INTRODUCTION

Female sex is a strong, independent risk factor for mortality after cardiac surgery, and it is included in the Society of Thoracic Surgeons [1] and EuroSCORE II risk models [2]. Yet, women are historically under-represented in cardiovascular trials, and analyses of registry data [3] suggest that, compared to men, women are less likely to undergo valvular surgery and have worse outcomes [3].

Minimally invasive cardiac surgery (MICS) for aortic or mitral repair or replacement is thought to produce better postoperative outcomes compared to conventional sternotomy (ST), including reduced blood loss, shorter hospital stays and higher patient satisfaction [4, 5]. On this premise, MICS may be particularly suited to patients who are at risk for surgery [6–8].

In this study, we hypothesized that MICS may be advantageous compared to conventional ST for female patients in terms of in-hospital mortality, operative parameters and postoperative complications. To this end, the goal of the study was to assess whether female sex as a risk factor for poor outcome after valve repair or replacement is a function of surgical approach (MICS or ST).

## MATERIALS AND METHODS

This study was an observational analysis of data from 9 cardiac units in Italy. Centre specifications are reported in the Supplementary Materials. The analysis included all patients who underwent isolated mitral (± tricuspid repair) or aortic valve surgery (repair and replacement) with ST or MICS from 31 December 2010 to 31 December 2019. Patients who required combined procedures (other than tricuspid repair) were excluded.

All centres entered cardiac surgery-related data into a common registry that was subject to review by a centralized clinical governance unit who checked data for accuracy and completeness on a monthly basis. In the present analysis, the primary outcome of interest was mortality defined as in-hospital mortality. Secondary outcomes included cardiopulmonary bypass (CPB) and cross-clamp (CC) times, postoperative renal failure, disabling stroke, need for blood transfusions, mechanical ventilation time and length of stay in the intensive care unit (ICU).

### Ethical statement

All patients provided written informed consent for the clinical and administrative storage of medical data. Given the observational nature of this study, the local ethics committee waived the need for patients’ consent to analyse the data from the registry.

### Minimally invasive surgical techniques

The decision to perform valve repair or replacement via a minimally invasive approach was based on the preference of the surgeon. All of the included cardiac units adopted a similar approach for minimally invasive aortic and mitral valve surgery as described previously [9, 10] and as reported in the Supplementary Materials. The types of valves implanted and the types of mitral valve repairs are also reported in the Supplementary Materials.

### Statistical analyses

Anonymized data were exported in a .csv sheet and uploaded in an Rstudio environment. Data distributions were checked for normality before further analysis with the Shapiro–Wilk test. Continuous data are presented as the mean and standard deviation (SD) or median and interquartile range. Unpaired *t*-tests or Wilcoxon rank sum tests were used for statistical comparisons. Categorical data are presented as proportions and were compared using the *χ*^2^ tests.

To balance the distribution of measured baseline covariates and given the observational nature of the data, we used a nearest neighbour 1:1 propensity score matching analysis (greedy matching) with a calliper of 0.2, based on the surgical approach (MICS or ST). We calculated standardized mean differences for each covariate in the propensity model to assess residual imbalances between the groups. A standardized mean difference of ±0.1 of SD units or less indicated an irrelevant difference.

Survival was analysed using the Kaplan–Meier method, and corresponding survival curves were built by plotting all observations. Comparisons of survival estimates for different patient strata were performed with the log-rank statistic.

A Cox proportional hazard regression model was constructed to identify factors associated with mortality and to assess the statistical interaction of the covariate ‘sex’ and ‘surgical approach’ on mortality in the general population. Covariates included in the regression model are specified in the Supplementary materials.

Risk factor distributions in each group were plotted using a random effects model and reported as risk ratios before and after matching. All statistical analyses were performed with RStudio Team (2020) (RStudio: Integrated Development for R. RStudio, PBC, Boston, MA, USA; URL http://www.rstudio.com/).

## RESULTS

### Baseline patient characteristics

**Figure 1:**
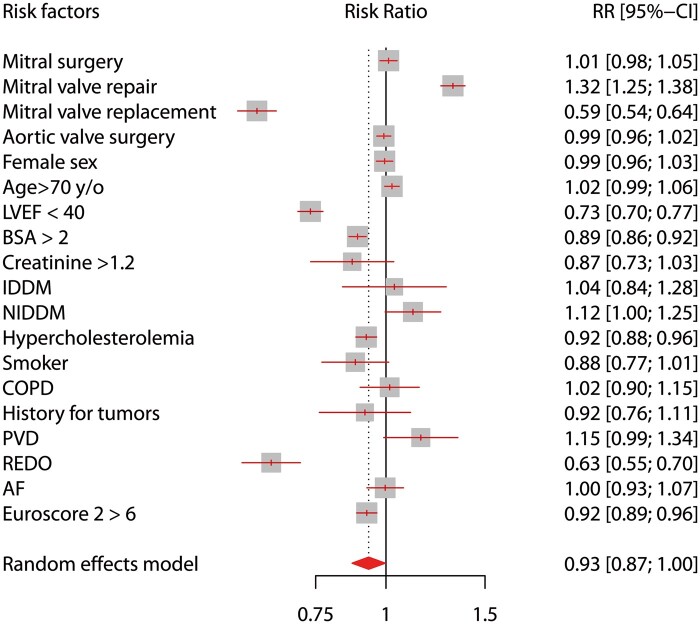
Whole cohort, forest plot, risk factor distribution among patients having minimally invasive cardiac surgery (MICS) and sternotomy. Data are reported as RR; keeping the MICS as the numerator, RR <1 indicates less prevalence of the risk factor and vice versa for RR >1. RR for confounding factors, MICS/sternotomy = 0.93; 95% CI 0.87–1. AF: atrial fibrillation; BSA: body surface area; CI: confidence interval; COPD: chronic obstructive pulmonary disease; IDDM: insulin-dependent diabetes mellitus; LVEF: left ventricular ejection fraction; NIDDM: non-insulin-dependent diabetes mellitus; PVD: peripheral vascular disease; RR: risk ratio.

A total of 15 155 patients underwent isolated valve surgery at 9 Italian cardiac centres during the study period; of these, 7674 patients underwent MICS (50.6%). Overall population baseline characteristics and those for female subpopulations are reported in Table 1. Proportions of female patients were similar between the MICS and ST groups (47.3% vs 47.6%, respectively; *P* = 0.76). Relevant differences in baseline characteristics are shown in Fig. 1 [risk ratio for confounding factors, MICS/ST = 0.93; 95% confidence interval (CI 0.87–1.0)]. The overall level of preoperative risk was higher in the ST group than in the MICS group [EuroSCORE II: 3.7 ± 6.4 (MICS) vs 4.0 ± 6.8 (ST); *P* = 0.005] and also higher for women in the ST group than for women in the MICS group [EuroSCORE II: 4.0 (SD: 6.8) vs 3.7 (SD: 6.4), respectively; *P* = 0.005] including among female patients only [ST vs MICS, 4.2 (SD: 6.3) vs 4.6 (SD: 6.9); *P* = 0.04]. The proportion of aortic and mitral valve procedures were similar between the MICS and ST groups (mitral, 45.2% vs 44.7%, *P* = 0.56; aortic surgery, 54.8% vs 55.2%, *P* = 0.75, respectively). However, more mitral valve repairs were performed in the MICS group whereas more associated tricuspid procedures were conducted in the ST group.

**Table 1:** Baseline patient characteristics

	Whole cohort (*N* = 15 155)		*P*-value	Female cohort (*N* = 7194)		*P*-value
	MICS [*N* = 7674 (50.6%)]	ST [*N* = 7481 (49.3%)]		Female sex: MICS [*N* = 3633 (50.5%)]	Female sex: ST [*N* = 3561 (49.4%)]	
Mitral surgery	3469 (45.2)	3346 (44.7)	0.56	1670 (46)	1700 (47.7)	0.13
Mitral valve repair	2619 (75.5)	1941 (58)	<0.001	1269 (76)	1020 (60)	<0.001
Mitral valve replacement	850 (24.5)	1405 (42)	<0.001	401 (24)	680 (40)	<0.001
Associated tricuspid repair	312 (9)	790 (23.6)	<0.001	167 (10)	425 (25)	<0.001
Aortic valve surgery	4205 (54.8)	4135 (55.2)	0.75	1963 (54)	1861 (52.2)	0.4
Female gender	3633 (47.3)	3561 (47.6)	0.84			
Age (years)	68.9 ± 12.2	68.4 ± 12.6	<0.001	71.3 ± 10.9	70.7 ± 11.3	0.01
BSA	1.81 ± 0.2	1.81 ± 0.19	0.51	1.7 ± 0.2	1.7 ± 0.1	0.22
LVEF (%)	54.7 ± 8.8	54.8 ± 8.9	0.58	55.3 ± 8.3	55.5 ± 8.2	0.28
Creatinine >1.2 mg/dl	243 (3.2)	272 (3.6)	0.12	101 (2.7)	79 (2.2)	0.1
Diabetes						
IDDM	172 (2.2)	162 (2.2)	0.75	91 (2.5)	72 (2.1)	0.1
NIDDM	590 (7.7)	515 (6.9)	0.07	313 (8.6)	250 (7)	0.01
Hypercholesterolaemia	2661 (34.7)	2812 (37.6)	0.01	665 (18.3)	580 (16.2)	0.05
Current smoker	471 (6.1)	410 (5.5)	0.1	113 (3.1)	155 (4.3)	<0.001
COPD	502 (6.5)	482 (6.4)	0.81	169 (4.7)	147 (4.1)	0.19
History of tumours	208 (2.7)	221 (3.0)	0.37	115 (3.2)	128 (3.6)	0.33
PVD	349 (4.5)	295 (3.9)	0.07	151 (4.2)	123 (3.5)	0.13
Redo surgery	413 (5.4)	644 (8.6)	<0.001	195 (5.4)	308 (8.6)	<0.001
AF^a^	1175 (15.3)	1149 (15.4)	0.9	694 (19.1)	635 (17.8)	0.24
EuroSCORE 2	3.7 ± 6.4	4 ± 6.8	0.005	4.2 ± 6.3	4.6 ± 6.9	0.04

Values are reported as mean ± SD or as number and frequency (%).

a Defined as all types of atrial fibrillation.

AF: atrial fibrillation; BSA: body surface area; COPD: chronic obstructive pulmonary disease; IDDM: insulin-dependent diabetes mellitus; LVEF: left ventricular ejection fraction; MICS: minimally invasive cardiac surgery; NIDDM: non-insulin-dependent diabetes mellitus; PVD: peripheral vascular disease; SD: standard deviation; ST: sternotomy.

### Operative details and postoperative course

**Figure 2:**
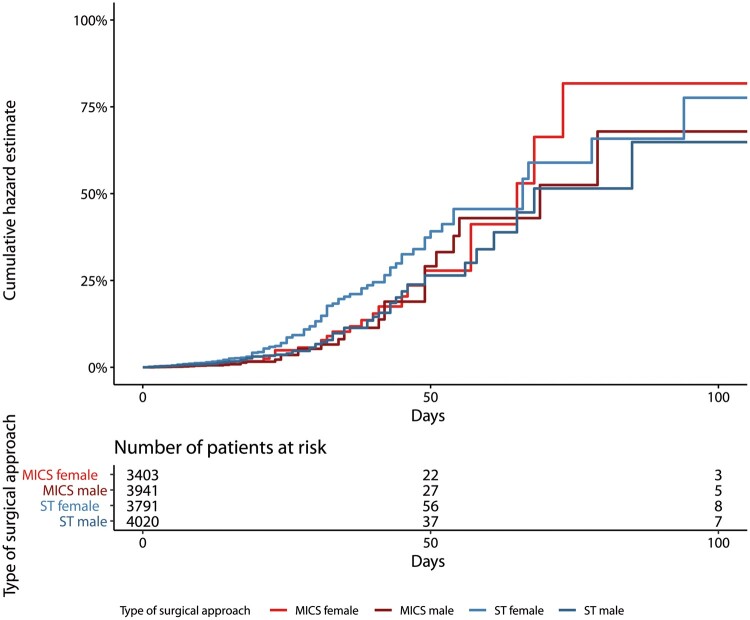
Whole cohort (*N* = 15 155), hazard estimate, MICS male/female and ST male/female [surgical approach and sex: hazard ratio 1.09 (95% confidence interval 0.7–1.7), *P* for interaction = 0.51]. MICS: minimally invasive cardiac surgery; ST: sternotomy.

Operative and postoperative details for the overall population (MICS vs ST) and female subpopulations are reported in Table 2. There were more favourable postoperative outcomes in the MICS group. Notably, postoperative mortality was significantly higher among female patients than among male patients in both groups (MICS: 2.4% vs 1.5%, *P* = 0.009 and ST: 2.8% vs 1.8%, *P* = 0.004) (Supplementary Material, Table S1). Postoperative mortality did not differ among female patients in the MICS and ST groups [2.4% vs 2.8%, respectively; hazard ratio (HR) 1.09, 95% CI 0.71–1.73; *P* (surgical approach × sex) = 0.51] (Fig. 2). Supplementary Material, Table S2 presents multivariable Cox regression models, with age (HR 1.05, 95% CI 1.01–1.23; *P* = 0.01), left ventricle function (HR 0.92, 95% CI 0.8–0.99; *P* = 0.01), female sex (HR 1.19, 95% CI 1.01–1.23; *P* = 0.01) and redo surgery (HR 1.11, 95% CI 1–1.51; *P* = 0.02) as covariates independently associated with postoperative mortality.

**Table 2:** Perioperative details

	Whole cohort (*N* = 15 155)		*P*-value	Female cohort (*N* = 7194)		*P*-value
	MICS [*N* = 7674 (50.6%)]	ST [*N* = 7481 (49.3%)]		Female sex: MICS [*N* = 3633 (50.5%)]	Female sex: ST [*N* = 3561 (49.4%)]	
CPB (min)	100.5 ± 71	86.3 ± 41.4	0.001	101 ± 35.8	82.3 ± 40.6	0.01
Cross-clamp (min)	88.9 ± 36.2	64.1 ± 20	0.001	84.6 ± 26.4	60.5 ± 28.5	<0.001
Ventilation time (h)	11.8 ± 47	13.6 ± 59.2	0.057	12.9 ± 51.2	15.2 ± 72.3	0.014
LOS ICU (days)	2.6 ± 5.3	2.8 ± 5.4	0.051	2.8 ± 5.2	3.05 ± 6.1	0.19
Blood transfusion^a^	3299 (42.9)	3467 (46.3)	0.01	1796 (49.4)	1866 (52.4)	0.15
Invalidating stroke	25 (0.3)	33 (0.4)	0.25	13 (0.4)	20 (0.6)	0.2
Renal failure	440 (5.7)	517 (6.9)	0.005	221 (6.1)	255 (7.2)	0.08
Hospital deaths	149 (1.9)	172 (2.3)	0.14	87 (2.4)	101 (2.8)	0.27

Values are reported as mean ± SD or number and frequency (%).

a Defined as at least 1 unit of blood transfused until discharge.

CPB: cardiopulmonary bypass; ICU: intensive care unit; LOS: length of stay; MICS: minimally invasive cardiac surgery; SD: standard deviation; ST: sternotomy.

CPB and CC times were longer in the female patient MICS group compared to the female patient ST group [CPB: 101 min (SD: 35.8 min) vs 82.4 min (SD: 40.6 min), *P* = 0.001 and CC: 84.6 min (SD: 26.4 min) vs 60.5 min (SD: 28.5), *P* < 0.001]. Ventilation time was shorter in the female patient MICS group compared with the female patient ST group [12.9 h (SD: 51.1 h) vs 15.2 h (SD: 72.3 h), *P* = 0.014]. Other postoperative variables were similar between female patients in the MICS and ST groups (Table 2).

### Propensity score matched cohort

**Figure 3:**
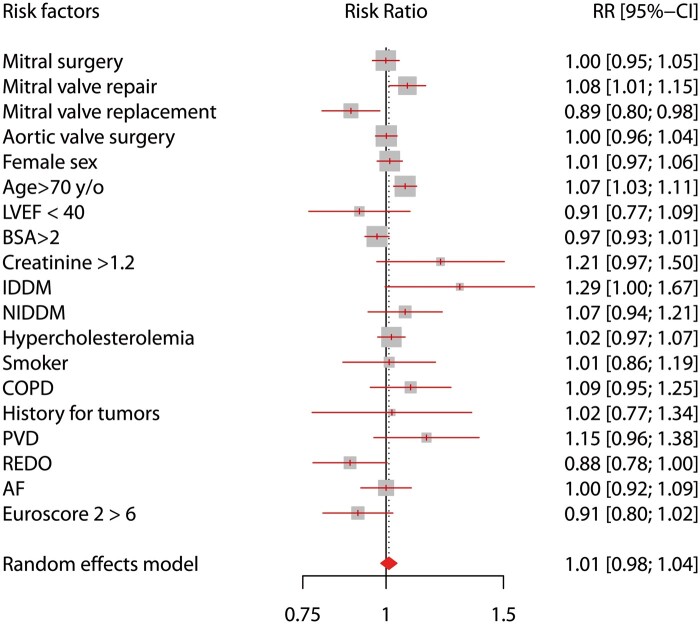
Propensity score matched forest plot, risk factor distribution among minimally invasive cardiac surgery (MICS) and sternotomy patients after propensity score matching analysis (*N* = 8970). Data are reported as RR; keeping the MICS as numerator, RR <1 indicates less prevalence of the risk factor and vice versa for RR >1. RR for confounding factors, MICS/sternotomy = 1.01; 95% CI 0.98–1.04. AF: atrial fibrillation; BSA: body surface area; CI: confidence interval; COPD: chronic obstructive pulmonary disease; IDDM: insulin-dependent diabetes mellitus; LVEF: left ventricular ejection fraction; NIDDM: non-insulin-dependent diabetes mellitus; PVD: peripheral vascular disease; RR: risk ratio.

Propensity score matching generated 4485 matched pairs for a total sample size of 8970 patients (Table 3). Of these 2144 (47.8%) female patients underwent MICS and 2117 (47.2%) female patients underwent ST. Baseline characteristics are reported in Fig. 3 (risk ratios for confounding factors, MICS/ST =1.01; 95% CI 0.98–1.04).

**Table 3:** Baseline patient characteristics with propensity score matching analysis

	Whole PSM cohort (*N* = 8970)		SMD	Female PSM cohort (*N* = 4261)		SMD
	MICS (*N* = 4485)	ST (*N* = 4485)		Female sex: MICS (*N* = 2144)	Female sex: ST (*N* = 2117)	
Mitral surgery	1996 (44.5)	1998 (44.5)	0.02	979 (45.7)	1049 (49.6)	0.07
Mitral valve repair	1397 (70)	1298 (65)	0.09	702 (71.7)	699 (66.6)	0.08
Mitral valve replacement	599 (30)	700 (35)	0.02	277 (28.3)	350 (33.4)	0.02
Aortic valve surgery	2489 (55.5)	2487 (55.4)	0.02	1165 (54.3)	1068 (50.4)	0.07
Female sex	2144 (47.8)	2117 (47.2)	0.01			
Age (years)	68.7 ± 12.2	68.25 ± 12.8	0.04	71.2 ± 10.9	70.6 ± 11.3	0.05
BSA	1.81 ± 0.21	1.81 ± 0.19	0.02	1.7 ± 0.2	1.7 ± 0.1	0.05
LVEF (%)	54.9 ± 8.86	54.7 ± 8.9	0.02	55.4 ± 8.4	55.3 ± 8.3	0.01
Creatinine >1.2 mg/dl	169 (3.8)	140 (3.1)	0.06	71 (3.3)	54 (2.6)	0.06
Diabetes						
IDDM	129 (2.9)	100 (2.2)	0.07	67 (3.1)	46 (2.2)	0.1
NIDDM	439 (9.8)	411 (9.1)	0.07	243 (11.3)	172 (8.1)	0.1
Hypercholesterolaemia	1971 (43.9)	1935 (43.1)	0.03	1034 (48.2)	997 (47.1)	0.03
Current smoker	283 (6.3)	280 (6.2)	0.05	81 (3.8)	100 (4.7)	0.05
COPD	381 (8.5)	350 (7.8)	0.1	129 (6)	89 (4.2)	0.09
History of tumours	160 (3.6)	162 (3.6)	0.03	93 (4.3)	92 (4.3)	0.07
PVD	238 (5.3)	207 (4.6)	0.03	106 (4.9)	96 (4.5)	0.03
Redo surgery	400 (8.9)	453 (10.1)	0.1	149 (6.9)	220 (10.4)	0.1
AF^a^	827 (18.4)	827 (18.4)	0.02	490 (22.9)	473 (22.3)	0.03
EuroSCORE 2	3.3 ± 6	3.8 ± 6.7	0.07	3.9 ± 6.5	4.4 ± 7.2	0.07

Values are reported as mean ± SD or number and frequency (%).

a Defined as all types of atrial fibrillation.

AF: atrial fibrillation; BSA: body surface area; COPD: chronic obstructive pulmonary disease; IDDM: insulin-dependent diabetes mellitus; LVEF: left ventricular ejection fraction; MICS: minimally invasive cardiac surgery; NIDDM: non-insulin-dependent diabetes mellitus; PVD: peripheral vascular disease; PSM: propensity score matching; SD: standard deviation; SMD: standardized mean difference; ST: sternotomy.

Operative and postoperative details are provided in Table 4.

**Table 4:** Perioperative details, propensity score matching analysis

	Whole PSM cohort (*N* = 8970)		*P*-value	Female PSM cohort (*N* = 4261)		*P*-value
	MICS (*N* = 4485)	ST (*N* = 4485)		Female sex: MICS (*N* = 2144)	Female sex: ST (*N* = 2117)	
CPB (min)	104.8 ± 35.6	86.3 ± 41.3	0.03	84.7 ± 34.2	82.4 ± 40.6	0.04
Cross-clamp (min)	88.1 ± 35.3	64.2 ± 30.1	0.04	64.1 ± 25.3	60.6 ± 28.5	<0.001
Ventilation time (h)	13.3 ± 54.3	12.4 ± 59.2	0.46	14.3 ± 54.3	14.2 ± 73.6	0.96
LOS ICU (days)	2.7 ± 4.9	2.7 ± 4.8	0.85	3 ± 5.4	2.9 ± 5.1	0.5
Blood transfusions^a^	1440 (32.1)	1649 (36.7)	0.001	881 (41)	864 (40.8)	0.9
Invalidating stroke	14 (0.3)	25 (0.6)	0.06	7 (0.3)	14 (0.7)	0.12
Renal failure	292 (6.5)	324 (7.2)	0.21	147 (6.9)	168 (7.9)	0.21
Hospital deaths	83 (1.9)	104 (2.3)	0.12	47 (2.2)	64 (3)	0.09

Values are reported as mean ± SD or number and frequency (%).

a Defined as at least 1 unit of blood transfused until discharge.

CPB: cardiopulmonary bypass; ICU: intensive care unit; LOS: length of stay; MICS: minimally invasive cardiac surgery; PSM: propensity score matching; SD: standard deviation; ST: sternotomy.

In-hospital mortality was not significantly different between female patients in the MICS and ST groups (2.2% vs 3%, respectively; *P* = 0.09) Postoperative variables including the need for blood transfusions, renal failure, disabling stroke, mechanical ventilation time and length of stay in the ICU were similar between female patients in the MICS and ST groups (Supplementary Material, TablesS3 and S4).

### Subgroup analysis: isolated aortic and mitral surgery

There were no significant statistical interactions between sex and surgical approach for in-hospital mortality in the aortic surgery [HR 0.79, 95% CI 0.37–1.7; *P* (surgical approach × sex) = 0.55] or mitral surgery subpopulations [HR 0.88, 95% CI 0.57–1.4; *P* (surgical approach × sex) = 0.59]. There were no differences in postoperative complications between female patients in the MICS and ST groups in either subpopulation (Supplementary Material, Table S4).

## DISCUSSION

This study found that female mortality rates did not differ for MICS and ST. Even after adjusting for baseline covariates, a MICS approach did not mitigate the influence of sex on in-hospital mortality or postoperative complications after valve surgery. The data do, however, suggest that MICS is not inferior to ST in terms of safety for female patients.

To the best of our knowledge, this is the first study to investigate the interaction of sex and surgical access on mortality and postoperative complications in the context of valve repair or replacement. Compared to men, women tend to have a higher burden of comorbidity conditions such as diabetes mellitus, hypertension, heart failure, cerebrovascular disease and anaemia. They also have smaller body surface area and generally are more ‘medically complex’ and frail than men [11]. Moreover, women with cardiovascular disease often experience a delay in diagnosis and treatment compared to men, which may partly explain the more advanced coronary or valvular disease at the time of surgery reported in the literature [12].

We have previously reported that MICS for valve repair or replacement is safe and effective including among high-risk patients [6]. Female sex is a factor included in most of the risk score computation methods for most of the risk scores [1, 2], especially for coronary artery bypass grafting surgery and combined/mixed cardiac procedures [11]. However, the exact role of sex in surgical risk for isolated valve procedures (repair and replacement) remains controversial.

In a nationwide study of all patients undergoing mitral valve surgery in the Netherlands between 2007 and 2011 (*N* = 3411 patients, 42% women), Mokhles *et al.* [13] reported no sex-specific differences in terms of early mortality; however, the study did not include any late outcomes. Chan *et al.* [14] analysed 743 Canadian patients (28% women) who underwent mitral valve repair for degenerative mitral regurgitation between 2001 and 2014 and similarly observed no specific sex differences in early and 5-year survival.

At the same time, a large study of Medicare beneficiaries (*N* = 183 792 patients) found significant sex-specific differences for early mortality (7.7% for women and 6.1% for men; *P* < 0.0001) after mitral surgery, repair and replacement; nevertheless, long-term survival was similar [15]. They also found that women were more likely to have their mitral valves replaced rather than repaired, and that repair restored life expectancy for men but not for women [15].

Kulik *et al.* [16] performed a retrospective analysis of 3118 patients (40.4% women) who had mitral or aortic valve replacement in Canada between 1976 and 2006 and found that women had worse long-term outcomes (including survival) after valve replacement surgery than men. Johnston *et al.* [11] reported that female sex did not influence early mortality; however, life expectancy after mitral valve repair was higher among men whereas that after mitral valve replacement was higher among women. The authors reported no sex differences with regard to aortic valve surgery.

In the Cardiothoracic Surgical Trial Network study that focused on secondary mitral regurgitation treated in 251 patients (38.2% women), women had higher mortality and worse quality of life after mitral surgery (both repair and replacement) than men [17]. A large cohort study of patients who underwent isolated left-sided valve surgery similarly found that women experienced higher in-hospital complications including all-cause mortality than men but that improvements in in-hospital clinical outcomes were observed over time for both sexes [18]. Seeburger *et al*. [19] also reported that men had better long-term survival than women after mitral valve repair.

Finally, with regard to isolated aortic valve replacement, findings from the OBSERVANT (Observational Study of Effectiveness of SAVR-TAVI Procedures for Severe Aortic Stenosis Treatment) trial showed that female sex was a risk factor for mortality and for postoperative blood transfusions [20].

Another important finding arising from our analysis was that the number of patients undergoing mitral valve repair was higher in the MICS group compared to the ST group, whereas tricuspid associated procedures were more frequent in the ST group. One potential explanation is that physicians elected to perform ST in more complex patients requiring replacement and associated tricuspid procedures. Nevertheless, female patients had the same likelihood of undergoing mitral valve repair as the general population (MICS: 76% vs 75.5% and 60% vs 58%, respectively).

### Limitations

Our study had several limitations. First, observed sex-specific differences in outcomes may be representative of surgical practice in Italy; accordingly, our findings require validation in other settings and countries to confirm their generalizability. Second, most isolated mitral valve procedures were valve repairs, and we did not differentiate between primary and secondary mitral valve regurgitation. Neither did we explore the influence of valve type (e.g. mechanical, tissue) on outcomes. The number of sutureless valves implanted was extremely low (<1%), such that it may be hypothesized that increased use of sutureless valves in a MICS context would result in better postoperative outcomes [21]. Third, the use of both ministernotomy and minithoracotomy approaches for aortic valve replacement in our study [9] may have produced a degree of clinical heterogeneity. Some of the baseline patient characteristics (i.e. history of coronary artery disease, need for rethoracotomy or conversion to ST) were not included in the analysis. Follow-up data were not recorded. Importantly, such findings should be interpreted also in the light of newer technologies such as transcatheter approaches.

Lastly, the study was subject to some bias and confounding because it was an observational study.

## CONCLUSION

Being female was associated with higher mortality in patients undergoing valve surgery. Minimal access cardiac surgery in female patients was safe; however, it did not provide any benefit in terms of reduced mortality or postoperative complications compared to standard ST.

## SUPPLEMENTARY MATERIAL

Supplementary material is available at *EJCTS* online.

## Supplementary Material

ezab369_Supplementary_Data

## ABBREVIATIONS

CPB: Cardiopulmonary bypass
CI: Confidence interval
HR: Hazard ratio
ICU: Intensive care unit
MICS: Minimally invasive cardiac surgery
SD: Standard deviation
ST: Sternotomy
