# Supplementary material for: Sex-specific differences and postoperative outcomes of minimally invasive and sternotomy valve surgery
Source: Eur J Cardiothorac Surg. 2021 Aug 15;61(3):695–702. doi: 10.1093/ejcts/ezab369 (PMC8858592; doi:10.1093/ejcts/ezab369)
Supplement: ezab369_Supplementary_Data [file ezab369_supplementary_data.zip › Moscarelli_Suppl material.pdf]

Supplementary material.

a) Participating centers

b) Flow chart inclusion exclusion criteria

c) Minimally invasive aortic surgical techniques

d) Minimally invasive mitral surgical techniques

e) Uni/multivariable analysis

a) Participating centers:

1. Anthea Hospital, Dept of cardiovasulcar surgery. Bari, Italy.
2. Citta' di Lecce Hospital, Dept of cardiovasulcar surgery, Lecce, Italy.
3. Maria Eleonora Hospital, Dept of cardiac surgery, Palermo, Italy.
4. Maria Cecilia Hospital, Dept of cardiovascular surgery, Lugo, Ravenna, Italy.
5. Villa Torri Hospital, Dept of cardiac surgery, Bologna, Italy.
6. Villa Azzurra Hospital (ICLAS) , Dept of cardiac surgery, Rapallo, Italy.
7. Maria Pia Hospital, Dept of cardiac surgery, Torino, Italy.
8. Salus Hospital, Dept of cardiac surgery, Reggio Emilia, Italy.
9. Monte Vergine Hospital, Dept of cardiac surgery, Avellino, Italy.

b) Inclusion exclusion criteria

Election to perform valve repair or replacement via minimally invasive approach was based on surgeons' preference, hence there were no pre-defined exclusion / inclusion criteria.

c) Minimally invasive aortic surgical techniques

Minimally invasive aortic is carried out through mini-sternotomy or mini-thoracothomy. The mini-sternotomy is performed in a j-shaped fashion up to the 3rd or 4th intercostal space; arterial and venous cannulation are carried out centrally through the main surgical site or through right femoral artery and vein. The right anterior mini-thoracotomy is performed through a 5-7 cm incision usually at the level of the second intercostal space; central or peripheral arterial and venous cannulation is achieved as described for the mini-sternotmy.

Sutureless (Perceval, Livanova, London, UK) or stented valve (mechanical prostheses: CarboMedics and Bicarbon aortic valves families, CarboMedics/LivaNova, London, United Kingdom; biological prostheses: porcine Hancock II and Mosaic™, Medtronic, Minneapolis, MN; pericardial: Carpentier-Edwards, Edwards Lifesciences, Irvine, CA) were used according to surgeons' preference. Isolated aortic valve repair procedures were also included.

d) Minimally invasive mitral surgical techniques

In all patients, the surgical approach is carried out through a 5-cm right anterolateral thoracotomy at the level of the third intercostal space. Rib spreading is limited, using a soft tissue retractor. A right femoral perfusion technique is used in most of the cases.

Arterial cannulation is performed with a femoral cannula (Bio-Medicus, Medtronic, Minneapolis, MN), and venous cannulation is accomplished with percutaneous cannulation of the femoral vein (Sorin LivaNova, London, United Kingdom) and jugular vein (Bio-Medicus, Medtronic). All cannulas are advanced into place using the Seldinger technique and are guided by transesophageal echocardiography. Two working ports are made for camera insertion (30 degrees, Karl Storz, Tuttlingen, Germany) and for carbon dioxide inflation as well as pump suctioning. A vacuum assist is used to optimize the venous return in all cases. Direct aortic cross-clamp is applied.

Several techniques were used for mitral valve repair, usually a combination of annuloplasty, leaflet resection and sliding, placement of neochordae and repair of the commissures. In valve replacement cases, standard stented mitral valve prostheses were implanted (mechanical prostheses: CarboMedics and Bicarbon aortic valves families, CarboMedics/LivaNova, London, United Kingdom; biological pros- theses: porcine Hancock II and MosaicTM, Medtronic, Minneapolis, MN; pericardial: Carpentier-Edwards, Edwards Lifesciences, Irvine, CA).

All patients receive normothermic antegrade blood cardioplegia

e) Uni/multivariable analysis. Covariates included.

A Cox proportional hazard regression model was planned to assess the interaction of the covariate ‘sex’ and ‘surgical approach’ on early mortality, using the general population and the propensity-score matching; univariable models were generated for the following prespecified confounder

- Surgery type (aortic / mitral), both repair and replacement (categorical variable)
- Surgical approach (ST / MICS) (categorical variable)
- Sex (female / male) (categorical variable)
- Age (continuous variable)
- Body surface area (continuous variable)
- Left ventricle ejection fraction (continuous variable)
- Creatinine (ordinal variable)
- Diabetes (ordinal variable)
- Hypercholesterolemia (ordinal variable)
- Current smoker (ordinal variable)
- COPD (ordinal variable)
- History for tumors (ordinal variable)
- PVD (ordinal variable)
- REDO surgery (ordinal variable)
- AF (ordinal variable)
- Euroscore 2 (continuous variable)

Statistically significant univariable predictors of mortality were included in the full multivariable model
