# Supplementary material for: Sex-specific differences and postoperative outcomes of minimally invasive and sternotomy valve surgery
Source: Eur J Cardiothorac Surg. 2021 Aug 15;61(3):695–702. doi: 10.1093/ejcts/ezab369 (PMC8858592; doi:10.1093/ejcts/ezab369)
Supplement: ezab369_Supplementary_Data [file ezab369_supplementary_data.zip › Moscarelli_Suppl tables.pdf]

Supplementary table 1.

	MICS		p-value	ST		p-value
	Female N=3633	Male N=4041		Female N=3561	Male N=3920	
CPB, min, mean±SD	101 (35.8)	91.2 (39)	P=0.36	82.3(40.6)	90(41.8)	P<0.001
Cross Clamp, min, mean±SD	84.6 (26.4)	92 (23.2)	P=0.42	60.5(28.5)	67.5(30.9)	P<0.001
Ventilation time, h, mean±SD	12.9(51.2)	11.4(44.3)	P=0.16	15.2(72.3)	12.1(43.8)	P=0.04
LOS ICU, days, mean±SD	2.8 (5.2)	2.8 (5.4)	P=0.92	3 (6.1)	2.6(6.1)	P=0.007
Blood transfusion, n (%)	1311(36)	876 (21.6)	P=0.001	1248 (35)	874 (22.2)	P<0.001
Invalidating stroke, n (%)	13 (0.3)	12 (0.3)	P=0.79	20 (0.5)	13 (0.3)	P=0.18
Renal failure, n (%)	221 (6)	219 (5.4)	P=0.25	255 (7.2)	262 (6.7)	P=0.47
Early mortality, n (%)	87 (2.4)	62 (1.5)	P=0.009	101(2.8)	71 (1.8)	P=0.004

Value are reported as mean±SD or number and frequency (%)

CPB: Cardiopulmonary bypass. LOS: Length of stay. MICS: Minimally invasive cardiac surgery. ST: Sternotomy.

§ Defined as at least 1 unit of blood transfused, until discharge.

Supplementary table 2. Uni-/multivariable (Overall population, N=15155)

Clinical variable	Crude HR 95% CI	p-value	Adj HR 95% CI	p-value
Surgery type (aortic/mitral)	1.21 (0.41, 1.9)	0.12	1.91 (0.8, 1.91)	0.92
ST/MICS	0.62 (0.33, 1.9)	0.92	0.44 (0.14, 1.12)	0.76
Sex female	**1.01 (1, 1.31)**	**0.01**	**1.19 (1.01,1.23)**	**0.01**
Age	**1.02 (1, 1.5)**	**0.01**	**1.05 (1.03, 1.07)**	**0.01**
BSA	0.35 (0.1, 1.21)	0.09	0.45 (0.22, 2.2)	0.51
LVEF	**0.8 (0.61, 0.92)**	**0.02**	**0.92 (0.8, 0.99)**	**0.02**
Creatinine > 1.2 mg/dl, n (%)	1.31 (0.21, 2.9)	0.14	0.77 (0.66, 2.92)	0.91
Diabetes	0.52 (0.23, 3.9)	0.11	0.24 (0.11, 2.12)	0.66
Hypercholesterolemia	0.45 (0.11, 3.22)	0.81	0.35 (0.21, 3.2)	0.72
Current smoker	0.75 (0.42, 2.77)	0.65	1.45 (0.72, 4.2)	0.81
COPD	1.35 (0.99, 4.21)	0.11	1.55 (1.22, 2.25)	0.75
History for tumors	1.22 (0.75, 2.41)	0.82	1.45 (1.22, 3.11)	0.91
PVD	0.45 (0.11, 3.71)	0.56	0.45 (0.22, 3.61)	0.61
REDO surgery	**1.05 (1, 1.41)**	**0.02**	**1.11 (1, 1.51)**	**0.02**
AF[§]	1.22 (0.99, 1.77	0.87	1.45 (1.22, 4.21)	0.58
Euroscore 2	1.1 (0.4, 3.9)	0.65	0.9 (0.4, 2,1)	0.72
Sex (F/M)*Approach(ST/MICS)[¶]	-	-	1.09 (0.71, 1.73)	0.51

AF: Atrial fibrillation. BSA: Body surface area. CI: Confidence interval; COPD: Chronic obstructive pulmonary disease. HR: Hazard Ratio; IDDM: Insulin dependent diabetes mellitus. LVEF: Left ventricular ejection fraction. MICS: Minimally invasive cardiac surgery. NIDDM: Non-insulin dependent diabetes mellitus. PVD: Peripheral vascular disease. ST: Sternotomy

[§]Defined as all type of atrial fibrillation

[¶]Indicates interaction

Bold denotes significance

Supplementary table 3. Uni-/multivariable (PSM cohort, N=8970)

Clinical variable	Crude HR 95% CI	p-value	Adj HR 95% CI	p-value
Surgery type (aortic/mitral)	1.21 (0.51, 1.8)	0.17	1 (0.8, 2.11)	0.91
ST/MICS	0.72 (0.43, 2)	0.9	0.51 (0.22, 1.11)	0.66
Sex female	**1.01 (1, 1.4)**	**0.01**	**1.2 (1, 1.3)**	**0.01**
Age	**1.01 (1, 1.6)**	**0.01**	**1.05 (1.02, 1.08)**	**0.01**
BSA	0.45 (0.21, 2)	0.89	0.5 (0.32, 2.1)	0.55
LVEF	**0.7 (0.5, 0.82)**	**0.01**	**0.9 (0.8, 0.99)**	**0.01**
Creatinine > 1.2 mg/dl, n (%)	1.21 (0.31, 3.1)	0.2	0.87 (0.56, 3)	0.81
Diabetes	0.32 (0.23, 3.1)	0.21	0.34 (0.21, 3.11)	0.76
Hypercholesterolemia	0.35 (0.21, 3.1)	0.9	0.45 (0.31, 3.21)	0.82
Current smoker	0.65 (0.32, 2.6)	0.66	1.35 (0.77, 4.1)	0.82
COPD	1.25 (0.89, 3.22)	0.21	1.45 (1.21, 2.35)	0.85
History for tumors	1.32 (0.85, 2)	0.92	1.35 (1.2, 2.1)	0.81
PVD	0.35 (0.21, 2.71)	0.66	0.66 (0.32, 2.62)	0.66
REDO surgery	1.15 (0.99, 1.51)	0.09	1.11 (0.88, 2.51)	0.78
AF[§]	1.11 (0.98, 1.67)	0.77	1.35 (1.21, 3.212	0.57
Euroscore 2	1.1 (0.4, 3.9)	0.65	0.9 (0.4, 2,1)	0.72
Sex (F/M)*Approach(ST/MICS)[¶]	-	-	0.95 (0.52, 1.72)	0.48

AF: Atrial fibrillation. BSA: Body surface area. CI: Confidence interval; COPD: Chronic obstructive pulmonary disease. HR: Hazard Ratio; IDDM: Insulin dependent diabetes mellitus. LVEF: Left ventricular ejection fraction. MICS: Minimally invasive cardiac surgery. NIDDM: Non-insulin dependent diabetes mellitus. PVD: Peripheral vascular disease. ST: Sternotomy

[§]Defined as all type of atrial fibrillation

[¶]Indicates interaction

Bold denotes significance

Supplementary table 4. Perioperative details. PSM subgroup analysis. Female aortic and mitral.

Female aortic N= 2233	MICS N=1165	ST N=1068	p-value
CPB, min, mean±SD	79.1 (27.8)	76.2 (36.4)	0.03
Cross Clamp, min, mean±SD	62 (20.1)	57.8 (24.8)	<0.001
Ventilation time, h, mean±SD	11.7 (28.5)	12.7 (47.6)	0.53
LOS ICU, days, mean±SD	2.7 (4.4)	2.9 (5.7)	0.46
Blood transfusion, n (%)	509 (43.6)	445 (41.7)	0.54
Invalidating stroke, n (%)	4 (0.3)	6 (0.6)	0.44
Renal failure, n (%)	72 (6.2)	80 (7.5)	0.25
Early mortality, n (%)	21 (1.8)	31 (2.9)	0.09
**Female mitral N=2028**	**Female Sex: MICS N= 979**	**Female Sex: ST N= 1049**	**p-value**
CPB, min, mean±SD	91.4 (39.6)	88.5 (43.4)	0.12
Cross Clamp, min, mean±SD	66.6 (30.2)	63.4 (31.5)	0.02
Ventilation time, h, mean±SD	17.4 (74.3)	15.6 (92.6)	0.6
LOS ICU, days, mean±SD	3.3 (6.5)	2.9 (4.4)	0.08
Blood transfusion, n (%)[§]	372 (37.9)	419 (39.9)	0.55
Invalidating stroke, n (%)	3 (0.3)	8 (0.8)	0.16
Renal failure, n (%)	75 (7.7)	88 (8.4)	0.57
Early mortality, n (%)	26 (2.7)	33 (3.1)	0.52

Value are reported as mean±SD or median and IQR, ore number and frequency (%)
CPB: Cardiopulmonary bypass. LOS: Length of stay. MICS: Minimally invasive cardiac surgery. PSM: Propensity score matching. ST: Sternotomy. TIA: Transient ischemic attack.
[§] Defined as at least 1 unit of blood transfused, until discharge.
